# Social media use, online experiences, and loneliness among young adults: A cohort study

**DOI:** 10.1111/nyas.15370

**Published:** 2025-05-11

**Authors:** Timothy Matthews, Louise Arseneault, Bridget T. Bryan, Helen L. Fisher, Rebecca Gray, Joanne Henchy, Terrie E. Moffitt, Candice L. Odgers

**Affiliations:** ^1^ School of Human Sciences, Faculty of Education, Health and Human Sciences University of Greenwich London UK; ^2^ King's College London, Social Genetic and Developmental Psychiatry Centre Institute of Psychiatry, Psychology & Neuroscience London UK; ^3^ ESRC Centre for Society and Mental Health King's College London London UK; ^4^ Department of Psychology and Neuroscience Duke University Durham North Carolina USA; ^5^ Duke University Population Research Institute Duke University Durham North Carolina USA; ^6^ Department of Psychiatry and Behavioral Sciences Duke University Durham North Carolina USA; ^7^ PROMENTA, Department of Psychology University of Oslo Oslo Norway; ^8^ Department of Psychological Science University of California Irvine California USA; ^9^ Social and Behavioral Sciences Gateway Irvine California USA

**Keywords:** COVID‐19, cybervictimization, digital technology, loneliness, social media, young adulthood

## Abstract

This study investigated patterns of digital technology use and their associations with loneliness in a cohort of 1632 young adults (mean age 26) in the UK who had been followed prospectively since childhood by the Environmental Risk Longitudinal Twin Study. Data were collected via an online survey in 2019–2020. Although overall time spent online was associated with greater loneliness, this was not the case for social media usage specifically. Use of social media platforms (e.g., Facebook, Instagram, Twitter) showed no association with loneliness. Instead, greater loneliness was associated with the use of Reddit and dating apps, while the use of WhatsApp was associated with lower loneliness. However, individuals who reported more compulsive use of digital technology, or experiences of online victimization, were lonelier on average, suggesting that the types of experiences individuals encounter online may be more related to loneliness than using particular platforms per se. Associations were robust to controls for a prior history of depression or anxiety at age 18. Moreover, findings remained broadly consistent between those who participated before versus during COVID‐19 lockdown measures. An exception was that certain types of media characterized by passive consumption were associated with loneliness prior to, but not during lockdown.

## Graphical abstract

This study investigated associations between digital technology use and loneliness in an online survey of 1632 young adults in the UK. Total time spent online was associated with greater loneliness; however, use of social media specifically was not. Problematic technology use and experiences of victimization online were also associated with greater loneliness. Findings were broadly consistent between participants completing the survey before versus during the COVID‐19 pandemic.

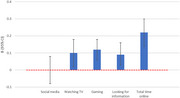

## INTRODUCTION

Advances in digital technology have transformed the ways in which people cultivate and maintain their relationships with each other in recent decades. In 2021, more than six billion smartphone subscriptions were held worldwide, a near‐twofold increase over the preceding 5 years.^1^ In the United States, online dating has become the preferred means of meeting romantic partners.^2^ During the COVID‐19 pandemic, video conferencing software became a vital, if imperfect tool for maintaining contact with friends and loved ones under lockdown conditions.^3^ As the range of modalities in which communication takes place has diversified, language itself has become embellished with new, digital forms of graphic symbolism: in 2015, Oxford Dictionaries selected an emoji as its word of the year for the first time.^4^

In tandem with these rapid technological changes and the ubiquitous role of digital communication in modern society, concerns have been raised about high levels of loneliness among adolescents and young adults,^5^ and whether life online may be contributing to young people's experiences of loneliness.^6^ Individuals born around the turn of the millennium represent the first generation to have grown up as digital natives. Furthermore, adolescents and young adults are also more likely to report that they often feel lonely, relative to older adults.^7^, ^8^ Some have argued that this generation's use of social media, rather than facilitating social connection, could encourage maladaptive, distant, superficial, or unrewarding ways of interacting, potentially driving feelings of loneliness due to a lack of satisfaction with these digital interactions.^9^, ^10^

The *displacement* hypothesis argues that the use of digital technology occupies time that could otherwise be spent engaging in offline social contact, which is argued to be more beneficial at reducing loneliness than more trivial online interactions.^11^ By contrast, the *stimulation* hypothesis argues that young people often use digital technology to build social connection and to enhance existing friendships that exist offline.^12^ An implication of this is that individuals highest in social resources offline could glean the most benefits from social media (the *rich get richer* hypothesis).^13^ However, an alternative perspective is that individuals already experiencing loneliness or associated difficulties may find online platforms a safe environment in which to fulfill their social needs and compensate for deficits in offline social connection.^14^ Other theoretical perspectives argue that digital technology could be a force either for good or bad with regard to loneliness, depending on contextual factors and the nature of the individual's engagement with different online platforms.^15^

Hence, to infer a straightforward linear association between the use of smartphones or social media and feelings of loneliness may be an oversimplification.^16^ Studies of depression, a strong correlate of loneliness in young people,^17^ indicate that increases in symptoms are associated not with the frequency of social media use, but with how and for what purpose it is used.^18^ Social media provides opportunities to access social support and enhance relationships that also exist offline.^19^ Individuals who use social media to build social connection in this way experience reductions in loneliness, whereas increases tend to be observed in those who resort to digital media as a coping mechanism.^20^, ^21^ Similarly, individuals who engage with digital technology in a compulsive manner, or in ways that interfere with their day‐to‐day responsibilities, may be more vulnerable to loneliness.^17^

Further ambiguity is introduced when social media is treated as a blanket term, without consideration of how different online platforms vary in their dynamics.^22^ For instance, some social media platforms are networking‐oriented, with a focus on interacting with friends and cataloging shared activities (e.g., Facebook).^23^ Others emphasize content creation, with the individual user cultivating a personal brand or aesthetic in front of an audience, with attendant pressures to impress, live up to expectations, and potentially generate revenue through brand partnerships (e.g., Instagram).^24^ Others still are dedicated to passive consumption of content, rather than communications (e.g., YouTube). In view of this, it is plausible that 4 h speaking to friends on Facebook Messenger could have quite different implications for loneliness compared to 4 h spent trying to perfect an Instagram story or watching videos on YouTube. Meanwhile, longer‐established forms of online communication such as blogs and discussion forums, and more specialized ones such as dating apps, represent further distinct forms of online communication. These may include a higher proportion of interactions with people who are not already known to the individual, in contrast to networking‐focused platforms that are also used to communicate with offline acquaintances. Hence, there is value in examining patterns of loneliness across different individual platforms, rather than conflating them.^25^, ^26^

The types of interpersonal encounters young people experience online could also be important determinants of how technology use impacts feelings of loneliness. Users who limit their networks to trusted individuals whom they also know offline might be expected to have a different experience from those who find themselves in less curated and structured online scenarios.^27^ For instance, some online spaces, such as messaging‐based platforms with limited oversight, may carry an increased risk of hostile or threatening encounters. Previous research has identified cybervictimization in adolescence as a salient risk factor for loneliness.^28^ This is a broad category of victimization that can include threats, public humiliation, sexual harassment, and invasion of privacy, and it could also have consequences that spill over into offline life.^29^ Though it frequently co‐occurs with offline peer victimization, cybervictimization is distinct in that it takes place outside any physical locus, the perpetrator may be anonymous, and the victimization may continue even if the victim logs off. A detailed examination of what particular types of online encounters are particularly salient for loneliness is warranted.^30^, ^31^

A further consideration is that social distancing rules imposed by many national governments in response to the COVID‐19 pandemic created an unprecedented scenario in which virtually all social contact beyond one's own household was moved onto digital platforms. This was a critical test of technology's ability (or inadequacy) to satisfy the social needs of the population. Young people reported increases in feelings of loneliness during lockdown^32^, ^33^, ^34^ and reported using social media as a means of coping with these feelings.^35^ While access to digital modes of communication was arguably a valuable lifeline, the case was also made that interaction via a screen could not adequately replace the richness of face‐to‐face contact and physical touch.^36^, ^37^ Moreover, lockdown may have had a leveling effect on associations between social media use and loneliness: patterns of usage that previously differentiated lonely from nonlonely individuals may not have done so at a time when the entire population had drastically fewer activities with which to fill their leisure time.^38^

In the present study, we investigate loneliness and use of digital technology among a cohort of young adults (aged 24–26) in the United Kingdom who had been followed from birth into their mid‐20s. We explore how patterns and extent of usage across different online platforms and apps, as well as online experiences such as victimization, are associated with feelings of loneliness. Furthermore, we examine young people's own perceptions as to whether social media use has a positive, negative, or immaterial effect on their experiences of loneliness. We also leverage prior measures of loneliness, depression, and anxiety, collected when participants were aged 18, to examine whether associations between digital technology and loneliness are observed only among individuals with pre‐existing risk. Due to the onset of the COVID‐19 pandemic during data collection, we additionally examine whether participants’ reports of their experiences differ between those who took part before versus during lockdown conditions.

## MATERIALS AND METHODS

### Participants

Participants were members of the Environmental Risk (E‐Risk) Longitudinal Twin Study, which tracks the development of a birth cohort of 2232 British children. The sample was drawn from a larger birth register of twins born in England and Wales in 1994–1995.^39^ Full details about the sample are reported elsewhere.^40^ Briefly, the E‐Risk sample was constructed in 1999–2000, when 1116 families (93% of those eligible) with same‐sex 5‐year‐old twins participated in home‐visit assessments. This sample comprised 56% monozygotic (MZ) and 44% dizygotic (DZ) twin pairs; sex was evenly distributed within zygosity (49% male). Ninety percent of participants were of white ethnicity.

Families were recruited to represent the UK population with newborns in the 1990s, to ensure adequate numbers of children in disadvantaged homes and to avoid an excess of twins born to well‐educated women using assisted reproduction. The study sample represents the full range of socioeconomic (SES) conditions in Great Britain. The cohort was evenly distributed across England and Wales (Figure S1), and the cohort's neighborhoods represent the full range of SES conditions in Great Britain. As shown in Figure S2, E‐Risk Study families’ addresses are a near‐perfect match to the deciles of the UK government's 2015 Index of Multiple Deprivation, which ranks British neighborhoods in terms of relative deprivation at an area level of approximately 1500 residents; approximately 10% of the E‐Risk Study cohort fills each of the index's 10% bands, indicating that the cohort accurately represents the distribution of deprivation in the United Kingdom.

Follow‐up home visits were conducted when the children were aged 7 (98% participation), 10 (96%), 12 (96%), and at 18 years (93%). Home visits at ages 5, 7, 10, and 12 years included assessments with participants as well as their mother (or primary caretaker). The home visit at age 18 included interviews only with the participants. The Joint South London and Maudsley and the Institute of Psychiatry Research Ethics Committee approved each phase of the study. Parents gave informed consent and twins gave assent between 5 and 12 years and then informed consent at ages 18 and 26.

The primary data in this study are drawn from the Social Media and Social Mobility (SM2) survey, an online survey of the E‐Risk cohort conducted between 2019 and 2020, when participants were aged 24–26 (referred to hereafter as *age 26*, the mean age at participation). Pilot testing was conducted from June to November 2019, and recruitment of the full sample began in December 2019. All E‐Risk Study participants were invited to complete a web‐based survey taking approximately 15–20 min. Questions covered usage of social media and digital technology, interpersonal trust, political engagement, mental health, employment, and beliefs about social mobility. Following the emergence of the COVID‐19 pandemic midway through data collection, further items were added regarding participants’ experiences of the pandemic, adapted from the Coronavirus Health and Impact Survey.^41^ Completed surveys were received from 1632 E‐Risk Study participants, representing 73.1% of the original cohort and 76.6% of those who took part in the age‐18 home visits. To counteract nonrandom response rates, extra time and recruitment efforts were devoted to boosting participation among male and low‐SES study members. In the final sample, representation of these groups was comparable to that in previous E‐Risk assessments (42% male in SM2 vs. 47% in the original E‐Risk cohort, and 31% low‐SES vs. 33% in the original E‐Risk cohort).

### Measures

#### Loneliness

In the age 26 survey, loneliness was assessed using four items from the UCLA Loneliness Scale, Version 3:^42^ “How often do you feel you lack companionship?”, “How often do you feel left out?”, “How often do you feel isolated from others?”, and “How often do you feel alone?”. At the item level, 50–52% of participants reported “hardly ever” having any of these feelings (0); 35–40% reported them “sometimes” (1), and 9–13% reported “often” (2). Items were summed to create a scale from 0 to 8 (M = 2.43, SD = 2.27; Cronbach α = 0.84).

#### Use of digital media and online experiences

Participants were asked at age 26 how much time they spent using social media, watching TV, gaming, and looking for information online, as well as their total time spent online overall (1 = “none” to 8 = “7 h or more a day”). They were also asked whether they used a range of social media and digital platforms (Facebook, Instagram, Twitter, Snapchat, WhatsApp, YouTube, Reddit, and dating sites/apps) and, if they did use any of these, how frequently (1 = “less than once a month” to 6 = “more than 5 times a day”). These platforms were selected to match those considered to be the most widely used among young people at the time of data collection. The overlap in usage of different types of digital media and platforms is summarized in Tables S1–S3.

The survey included a standalone question asking participants’ beliefs about how social media made them feel: “more lonely,” “less lonely,” or “no difference.” Two further questions asked about the amount of time participants spent actively posting on social media, and passively scrolling without posting (1 = “never” to 7 = “more than 5 times a day”). Perceived compulsive or problematic technology use was assessed via a sum scale of 7 items such as “How often have you neglected work, family or friends because you are using technology?” (M = 10.90, SD = 2.88, Cronbach α = 0.76). The full list of items and response choices is provided in the Supporting Materials.

Participants were asked if they had experienced any of seven types of online victimization: being called offensive names (endorsed by 36% of participants), being bothered or harassed (33%), being physically threatened (13%), being purposely embarrassed (28%), being asked unwanted sexual questions (21%), having sexually explicit photos of themselves shared without consent (8%), and receiving sexually explicit photos without consent (27%). Responses were coded 1 (“Never”), 2 (“Once”), and 3 (“Multiple times”). These items were used to construct composite variables reflecting “any victimization at least once” (score of 2 for any type of victimization), “any repeated victimization” (score of 3 for any type of victimization), and “multiple victimization” (2 or more different types of repeated victimization).

#### Covariates

At the age 18 assessment, loneliness was assessed using the same 4‐item short version of the UCLA Loneliness Scale (M = 1.57, SD = 1.94, Cronbach α = 0.83). This was completed as part of a computer‐based self‐complete questionnaire. Age 18 assessments of the participants are included in the current analyses to test whether a prior history of loneliness explained or modified the associations observed. In addition, assessments of major depressive disorder and generalized anxiety disorder were conducted via a structured clinical interview, based on the diagnostic criteria in the Diagnostic and Statistical Manual of Mental Disorders, version 4.^43^ Due to the high co‐occurrence of loneliness with these disorders,^17^ these mental health symptoms/diagnoses were selected as covariates and potential moderators.

Additional covariates included the biological sex of the participants at birth as reported by their mothers, and the family's SES, measured via a composite of parental income, education, and occupation, measured when participants were aged 5. The three SES indicators were highly correlated (*r*’s = 0.57–0.67, *p*’s < 0.05) and loaded significantly onto one latent factor (factor loadings = 0.80, 0.70, and 0.83 for income, education, and occupation, respectively). The latent variable was categorized into tertiles; that is, low, medium, and high SES.^44^

### Data analysis

Analyses were conducted in Stata, version 16.^45^ Associations between loneliness and the use of social media and digital technology at age 26 were tested using a series of linear regressions, with loneliness as the dependent variable. Each predictor was entered individually in separate regression models, controlling for sex and SES. Where significant associations were observed, age 18 depression and anxiety were further controlled for. As an additional step in each analysis, interaction effects were tested between the independent variable and sex, age 18 loneliness, and age 18 depression and anxiety. Due to the nonindependence of observations in datasets of twins, all analyses used robust standard errors, obtained via the *vce cluster* command in Stata.^46^

### Potential impact of the COVID‐19 pandemic

Almost two‐thirds (60.1%) of surveys had been completed prior to March 23, 2020, the start date of the UK's first national lockdown in response to the COVID‐19 pandemic. Slightly less than one quarter (23.7%) of surveys were completed between this date and July 4, when hospitality venues were allowed to reopen, signifying a major relaxing of restrictions. A further 10.9% were returned during this period to September 14, when the *rule of six* was introduced to limit the size of social gatherings. The remaining 5.2% of surveys were returned in the 1‐month period thereafter (Figure S3). Consequently, moderation analyses were carried out for all of the associations tested, in order to determine whether they differed in direction or magnitude before versus during the pandemic. For the purpose of these analyses, the March 23 lockdown date was used as the cutoff to define the *before* and *during* subgroups. The demographic characteristics of these two groups are summarized in Table S4.

### Open science

The premise and data analysis plan for this project were preregistered online at https://sites.duke.edu/moffittcaspiprojects/forms/projects_2021/, and the analysis code is available at https://github.com/t‐matth/loneliness‐socialmedia.

## RESULTS

### Do young adults perceive social media as contributing to their feelings of loneliness?

The vast majority (71.4%) of participants reported that using social media made “no difference” to their feelings of loneliness. Among the rest of the sample, responses were distributed fairly evenly between those who believed it made them “less lonely” (12.8%) and those who believed it made them “more lonely” (15.9%). Participants who reported that social media made “no difference” to their loneliness at age 26 also had the lowest mean scores of loneliness at age 18 (M = 1.34), compared to those who reported that it made them feel “less lonely” (M = 1.92, *t*_(1592)_ = 3.47, *p* = 0.001) or “more lonely” (M = 2.48, *t*_(1592)_ = 7.39, *p* < 0.001).

### Is usage of different digital media associated with loneliness?

When looking at usage habits with different forms of digital media, loneliness was not associated with the amount of time spent on social media (Table 1). It was, however, associated with greater time spent watching TV, gaming, and looking for information online, and also with greater total time spent online in general. Individuals with a prior history of depression were also more likely to report spending more time online, and to report seeking out information online (Table S5); however, this did not explain the associations with loneliness. In addition, the association between TV watching and loneliness at age 26 was stronger among people who had been lonelier at age 18 (interaction term β = 0.07; 95% CI [0.02, 0.12]; *p* = 0.005). No other moderating effects of age 18 loneliness, depression, or anxiety were observed.

**TABLE 1:** Associations between usage of digital media and loneliness.

Time spent on digital media	Mean (SD)	Association with loneliness									
		Model 1					Model 2				
		B	95% CI	β	95% CI	*p*	B	95% CI	β	95% CI	*p*
Social media	5.09 (1.54)	0.00	−0.08, 0.08	0.00	−0.05, 0.06	0.951	0.00	−0.08,.08	0.00	−0.05, 0.05	0.974
Watching TV	5.19 (1.51)	0.10	0.02, 0.18	0.07	0.02, 0.12	**0.010**	0.10	0.02, 0.18	0.07	0.02, 0.12	**0.011**
Gaming	2.75 (2.08)	0.12	0.06, 0.18	0.11	0.05, 0.17	**<0.001**	0.11	0.04, 0.17	0.10	0.04, 0.16	**0.001**
Looking for information	3.95 (1.67)	0.09	0.03, 0.16	0.07	0.02, 0.12	**0.007**	0.07	0.00, 0.14	0.05	0.00, 0.10	**0.043**
Total time online	6.35 (1.44)	0.22	0.14, 0.30	0.14	0.09, 0.19	**<0.001**	0.20	0.11, 0.28	0.12	0.07, 0.18	**<0.001**

*Note*: Model 1: Associations adjusted for sex and socioeconomic status. Model 2: Associations adjusted further for age 18 major depressive disorder and generalized anxiety disorder. All analyses are adjusted for the nonindependence of twin observations. B = unstandardized regression coefficient. β = standardized regression coefficient. Significant *p*‐values are indicated in bold.

Abbreviation: CI, confidence interval.

Participants who used networking‐oriented platforms such as Facebook, Instagram, Twitter, and Snapchat were no more or less lonely than those who did not use them (Table 2). Users of WhatsApp were on average less lonely than individuals who did not use this app, and this association remained significant after controlling for diagnoses of depression and anxiety. Meanwhile, users of other types of digital platforms (described below) were lonelier, on average, than those who said they did not use these platforms. These platforms included websites oriented more toward passive consumption (YouTube), large content‐rating platforms (Reddit), and dating sites/apps. These associations were robust to when controlling for both depression and anxiety, except for the association with YouTube which became nonsignificant. No moderating effects of age 18 loneliness or mental health symptoms were detected.

**TABLE 2:** Associations between use of specific digital apps/platforms and loneliness.

	Association with loneliness										
Digital platforms used	% Yes (*N*)	Model 1					Model 2				
		B	95% CI	β	95% CI	*p*	B	95% CI	β	95% CI	*p*
Facebook	87.9 (1432)	−0.22	−.59,.15	−0.03	−0.09, 0.02	0.237	−0.10	−0.47, 0.27	−0.01	−0.07, 0.04	0.586
WhatsApp	86.7 (1412)	−0.42	−.79, −.04	−0.06	−0.12, −0.01	**0.028**	−0.38	−0.75, −0.01	−0.06	−0.11, 0.00	**0.046**
Instagram	77.5 (1263)	−0.28	−.58,.02	−0.05	−0.11, 0.00	0.071	−0.20	−0.51, 0.10	−0.04	−0.09, 0.02	0.182
YouTube	72.6 (1183)	0.29	.04,.55	0.06	0.01, 0.11	**0.023**	0.21	−0.05, 0.46	0.04	−0.01, 0.09	0.115
Snapchat	55.9 (910)	−0.07	−.31,.18	−0.01	−0.07, 0.04	0.593	−0.01	−0.25, 0.24	0.00	−0.06, 0.05	0.942
Twitter	31.6 (514)	−0.17	−.41,.08	−0.03	−0.08, 0.02	0.191	−0.15	−0.40, 0.09	−0.03	−0.08, 0.02	0.223
Reddit	13.3 (217)	0.72	.37, 1.08	0.11	0.06, 0.16	**< 0.001**	0.62	0.27, 0.97	0.09	0.04, 0.15	**0.001**
Dating sites/apps	12.0 (195)	1.19	.83, 1.55	0.17	0.12, 0.22	**< 0.001**	1.15	0.78, 1.52	0.16	0.11, 0.22	**< 0.001**

*Note*: Model 1: Associations adjusted for sex and socioeconomic status. Model 2: Associations adjusted further for age 18 major depressive disorder and generalized anxiety disorder. All analyses are adjusted for the nonindependence of twin observations. Column “% Yes (*N*)” refers to the number of participants who reported using each platform. B = unstandardized regression coefficient. β = standardized regression coefficient. Significant *p*‐values are indicated in bold.

Abbreviations: CI, confidence interval; *N*, number.

With regard to frequency of usage, among the specific eight types of online platforms that were tested, only YouTube showed an association with elevated loneliness, whereby individuals who reported spending more time on YouTube also reported higher levels of loneliness (β = 0.20; 95% CI [0.14, 0.26]; *p* < 0.001). Individuals with a prior history of depression and anxiety at age 18 were also more likely to report spending more time on YouTube (Table S5); however, these prior mental health problems did not explain the association with loneliness. Meanwhile, individuals who reported higher frequency use of WhatsApp reported lower feelings of loneliness (β = −0.20; 95% CI [−0.32, −0.08], *p* = 0.001). This association also survived controlling for depression and anxiety. For all other platforms and apps, frequency of use was not associated with loneliness, though a marginal nonsignificant trend was observed between more frequent Instagram use and lower loneliness (β = −0.05; 95% CI [0.11, 0.00]; *p* = 0.061). One moderating effect was observed, whereby greater frequency of Instagram use was associated with lower loneliness among participants who met diagnostic criteria for anxiety at age 18 (β = −0.24; 95% CI [−0.43, −0.05]; *p* = 0.014).

### Are online behaviors and experiences associated with loneliness?

When examining different ways of engaging with social media, neither actively posting nor passive scrolling were associated with loneliness (posting: β = −0.02; 95% CI [−0.08, 0.04]; *p* = 0.445; scrolling: β = −0.04; 95% CI [−0.10, 0.01]; *p* = 0.116). However, compulsive or problematic technology use was associated with increased loneliness (β = 0.37; 95% CI [0.32, 0.42]; *p* < 0.001). Moreover, individuals who reported using social media to improve their mental or physical health reported greater feelings of loneliness (β = 0.22; 95% CI [0.17, 0.27]; *p* < 0.001). These associations remained significant after controlling for prior depression and anxiety. Participants who reported feeling emotionally supported by others online did not deviate from the average in their feelings of loneliness. However, among those who met diagnostic criteria for anxiety at age 18, feeling emotionally supported was associated with lower loneliness at age 26 (β = −0.20; 95% CI [−0.38, −0.01]; *p* = 0.036). This was also the case among participants who had scored above the median for feelings of loneliness at age 18 (β = −0.08; 95% CI [−0.15, −0.01]; *p* = 0.023).

Participants who had experienced online victimization also reported greater loneliness (any victimization at least once: β = 0.22; 95% CI [0.17, 0.27]; *p* < 0.001; any repeated victimization: β = 0.22; 95% CI [0.17, 0.27]; *p* < 0.001). Exposure to two or more different types of repeated online victimization showed a similar magnitude of association (β = 0.21; 95% CI [0.16, 0.25]; *p* < 0.001). These associations were robust when controlling for age 18 depression and anxiety, and no moderating effects of these factors were observed.

### Do associations between technology use and loneliness differ by sex?

Females reported higher mean levels of loneliness than males (*t*_(1,591)_ = 2.15, *p* = 0.032). When stratifying analyses by sex, an association was observed in males between increased time spent on Twitter and lower feelings of loneliness (β = −0.15; 95% CI [−0.28, −0.01]; *p* = 0.040), whereas no association was observed in females (β = 0.05; 95% CI [−0.08, 0.18], *p* = 0.407). Meanwhile, experiencing repeated online victimization was more strongly associated with loneliness among females than males, though effects were significant for both sexes (males: β = 0.16; 95% CI [0.09, 0.24]; *p* < 0.001; females: β = 0.26; 95% CI [0.20, 0.33]; *p* < 0.001). No other sex differences were observed in the associations reported.

### Did associations between technology use and loneliness differ during the COVID‐19 pandemic?

**FIGURE 1:**
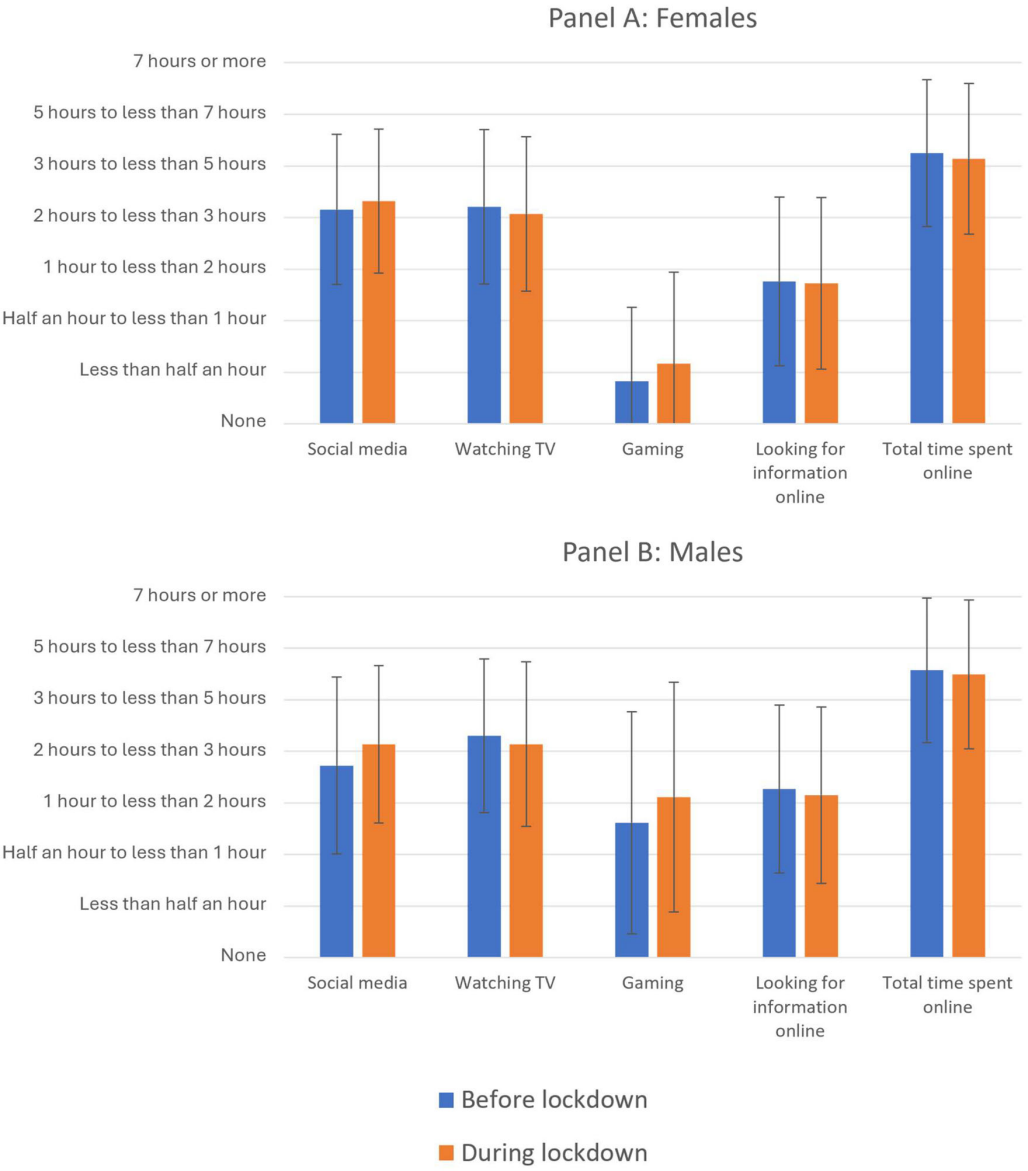
Time spent using digital media before and after the onset of COVID‐19 restrictions in the UK, stratified by biological sex. Error bars reflect ± 1 SD about the mean.

**FIGURE 2:**
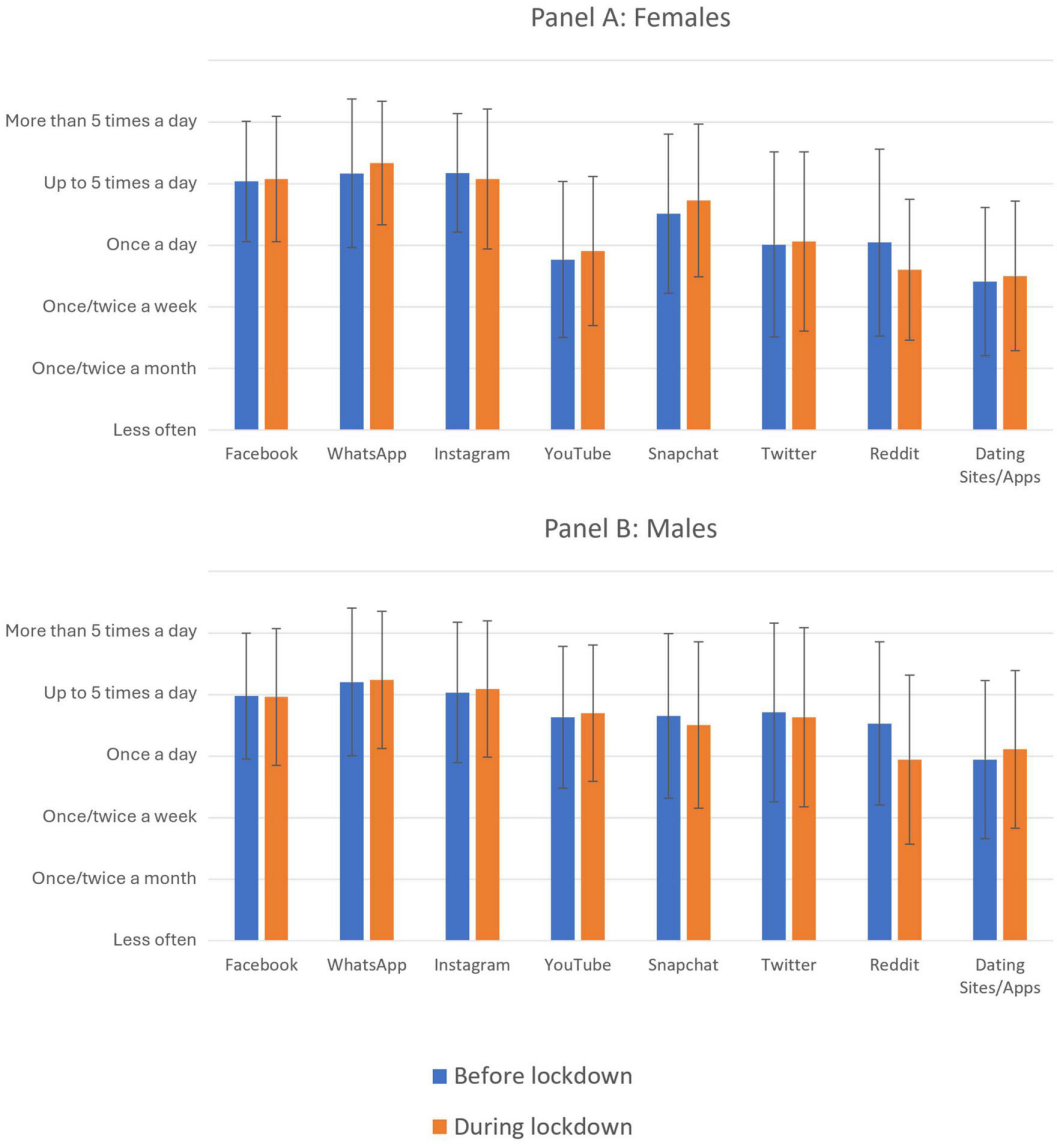
Frequency of use of social media platforms before and after the onset of COVID‐19 restrictions in the UK, stratified by biological sex. Bars reflect mean scores on a 6‐point Likert scale (1 = “Less often [than one/twice a month]”; 6 = “More than 5 times a day”). Error bars reflect ± 1 SD about the mean.

Overall patterns of technology use were comparable among participants who completed the survey during the pandemic versus those who completed it before (Figures 1 and 2). Further, mean levels of loneliness did not differ between these groups (before pandemic: M = 2.46, SD = 2.29; during pandemic: M = 2.39, SD = 2.24; *t*_(1591)_ = −0.59, *p* = 0.552). This was the case for both male and female participants, and for low, middle, and high SES groups.

When asked directly if they had felt more lonely since the onset of the pandemic, 13.4% of participants responded “yes,” 29.2% “somewhat,” and 48.7% “no,” with the remaining 8.8% responding “not applicable” or “don't know.” The proportion of people reporting that social media made them feel lonelier was slightly higher before the pandemic (18.1%), whereas during the pandemic, more people reported that it made them feel less lonely (14.3%).

Time spent watching TV was associated with greater loneliness prior to the pandemic (β = 0.09; 95% CI [0.02, 0.16]; *p* = 0.008), but not during it (β = 0.03; 95% CI [−0.05, 0.11]; *p* = 0.460). This was also the case for time spent seeking information online (before pandemic: β = 0.07; 95% CI [0.01, 0.14]; *p* = 0.022; during pandemic: β = 0.06; 95% CI [−0.02, 0.15]; *p* = 0.141). Using YouTube was also associated with greater loneliness before, but not during, the pandemic (before: β = 0.09; 95% CI [0.02, 0.15]; *p* = 0.011; during: β = 0.01; 95% CI [−0.07, 0.09]; *p* = 0.816), as was use of Reddit (before: β = 0.14; 95% CI [0.07, 0.20]; *p* < 0.001; during: β = 0.07; 95% CI [−0.01, 0.14]; *p* = 0.104). Meanwhile, increased frequency of Instagram use was associated with lower loneliness before the pandemic (β = −0.08; 95% CI [−0.16, −0.01]; *p* = 0.032), but not during it (β = −0.01; 95% CI [−0.10, 0.07]; *p* = 0.736).

## DISCUSSION

In the present study, we investigated whether young people who spend more time using various digital platforms also report higher levels of loneliness. We leveraged data from a large population‐based cohort with measures of participants’ past experiences of loneliness and mental health problems to also test whether individuals with mental health problems in adolescence exhibited stronger associations between social media use and loneliness, or went on to use social media in different ways. Overall, we found that while lonelier young people spent more time online overall, they did not spend more time on social media specifically. Moreover, the findings also showed that the association between time spent online and loneliness should not be interpreted at face value. Similar to other research that has illustrated digital technology can be a double‐edged sword for already vulnerable groups,^47^ we found that lonely individuals were more likely to spend that time seeking resources to support their mental and physical health, though at the same time, they were also more likely to report using digital technology in maladaptive and compulsive ways that interfered with their priorities and obligations. This indicates that lonely young people's digital media engagement, and the underpinning motivations for this engagement, cannot be summarized in simple terms; instead, it likely represents a diverse spectrum of individual circumstances.^48^

When looking specifically at social media, time spent using social media was not associated with loneliness, in contrast to consumption of other digital media such as TV and games. This is consistent with other research suggesting that concerns about the impact of social media on young people's well‐being may be overstated.^19^, ^49^, ^50^, ^51^, ^52^, ^53^ However, treating social media as a homogenous category appeared to obscure differences between the various types of platforms it subsumes as well as heterogeneity in individuals’ reasons for using these different platforms. Participants who used networking‐oriented platforms, through which they were likely to interact with people they also knew offline, did not report above‐average feelings of loneliness. In the case of WhatsApp, users reported feeling less lonely on average, perhaps because this app is designed to facilitate conversation between people who are also connected offline. The platforms that showed an association with greater loneliness had distinct features. For instance, YouTube primarily facilitates passive consumption of content, and the linear association of time spent on this platform with loneliness may indicate a selection effect, with lonelier individuals consuming greater amounts of video content as a means of coping. The higher rates of loneliness among users of Reddit may reflect a similar selection effect, whereby lonely individuals actively seek out the mental health support communities that exist on this platform.^54^

Although social media provides opportunities to build social connection and access support, individuals’ online behaviors and experiences also have the potential to be deleterious. Those who reported using social media and digital devices as a coping mechanism or in compulsive ways that interfered with their day‐to‐day tasks also reported elevated levels of loneliness. Though the direction of the association is unclear, it supports previous findings that patterns of technology use could be markers of risk for loneliness.^17^ Furthermore, the types of experiences a person encounters online can be positive or negative, with implications for feelings of loneliness.^55^ We have previously shown that cybervictimization in adolescence is a risk factor for loneliness at the age of 18.^28^ In this follow‐up, we find a similar association between these experiences among individuals in their mid‐20s, indicating that cybervictimization is not only a problem confined to adolescence.^56^

A recent meta‐analysis^49^ highlights that patterns of associations between social media and ill health can be influenced by the framing of questionnaire items: when individuals are asked to report specifically on the negative roles of social media in their lives (such as compulsive use), associations with psychological well‐being tend to be detected. Conversely, when asked to report on more neutral aspects of social media use, such as number of contacts or frequency of use, such associations are typically not detected. Our findings are consistent with this pattern, and underscore the importance of the framing of items when constructing measures of social media use and online behavior.

### Potential impact of the COVID‐19 pandemic

Despite the significant disruption to social activities brought about by the COVID‐19 pandemic, the data collected during lockdown did not differ substantially from the data collected beforehand, in terms of participants’ reported feelings of loneliness or digital habits. To the extent that differences were observed, it was generally the case that digital platforms which were associated with greater loneliness prior to the pandemic (such as TV and YouTube) were no longer associated with it after social distancing restrictions had been implemented. This could indicate a leveling effect, whereby the population in general was spending more time online than usual while in lockdown, and, therefore, online habits did not reliably differentiate lonely from nonlonely individuals during this period.

### Limitations

The small significant associations that were observed between loneliness and the use of specific digital platforms should be interpreted cautiously, in view of the observational nature of the study and the potential for confounding by third variables. A clear illustration of this is that the use of dating apps is most likely a proxy for being single, and feelings of loneliness may also be an indicator of this, thus explaining the correlation between the two. Similar considerations may hold true for other platforms: individuals whose feelings of loneliness arise from circumstances offline may self‐select into specific types of online spaces in order to cope with or alleviate these feelings. Therefore, a directional effect of social media usage on loneliness (or vice‐versa) cannot be inferred.

Some broader considerations with regard to the data collection and analyses should also be acknowledged. The SM2 survey was cross‐sectional in nature, and although pre‐existing data on some key variables were available, it was not possible to advance conclusions about the directionality or causality of the associations reported. Furthermore, self‐report measures of social media and technology use can be less reliable than more objective measures due to their subjectivity and potential for recall bias. Tracking actual usage data was beyond the scope of the present study; however, these data suggest that it would be a promising objective in future research.

In addition, while the standalone items about active posting and passive scrolling showed null associates with loneliness, they referred to participants’ social media usage in general and hence did not capture the heterogeneity of usage within individual platforms. While we observed that loneliness tended to be associated with platforms that were oriented toward passive consumption rather than networking, future research should consider how individuals vary both within and across platforms in their approach to social media. Similarly, the analyses on cybervictimization were not platform‐specific, and future research should aim to examine how it is experienced differently across online spaces with varied dynamics. However, as observed in this study, the types of digital media and platforms people engage with are not independent, and the usage of one platform is often correlated with the usage of one or more other platforms. A further challenge is that the landscape of social media is constantly evolving: new platforms can undergo a rapid ascendancy (such as TikTok, for which data was not collected at the time of this survey), and even existing platforms can undergo changes in policy or culture that shape how users interact with them.

The participants who completed the survey before versus during the pandemic are independent samples, and, therefore, it is not possible to make true pre and post comparisons. Moreover, it should be noted that while the data collection for this study overlapped with the first UK lockdown implemented in March 2020, it had concluded before the implementation of the second lockdown in November of the same year. Evidence from other studies suggests that although rates of loneliness among young people did not change substantially during the first UK lockdown, an uptick was observed during the second lockdown.^32^, ^57^ Therefore, these data are not necessarily illustrative of the impact of the pandemic as a whole, nor of lockdown restrictions, which varied significantly by country.

As the E‐Risk Study is a cohort of twins, all participants grew up with a sibling of the same age and biological sex as themselves, which may reduce feelings of loneliness on average compared to conventional sibling pairs or singletons. However, as twins enter adulthood and increasingly lead lives independently of each other, it would be expected that feelings of loneliness and the factors shaping these feelings would diverge similarly.

### Implications

Discussions around the putative harms of digital technology use have suggested that it may promote increased feelings of loneliness in young people, whether by displacing face‐to‐face social interaction or by encouraging social comparison or fear of missing out. These discussions have placed particular emphasis on the role of social media giants such as Facebook and Instagram. Consistent with previous research, the present study does not find support for these concerns in general, at least for people in their mid‐20s. Instead, the findings suggest that individuals who report engaging with digital technology in maladaptive ways or who experience victimization online may constitute already vulnerable groups toward whom concern may be better targeted.

## CONCLUSIONS

Amidst growing recognition of the high prevalence of loneliness in young people, social media has been posited as a contributing factor to this phenomenon. The present study indicates that social media use per se, and the frequency thereof, do not appear to signal an increased risk of loneliness. Instead, many popular platforms may provide opportunities to build and maintain social connections. Meanwhile, some other platforms, such as Reddit, appear to have characteristics that people who are already lonely are drawn to. Longitudinal and experimental work is required to test for causal links between the use of specific platforms and features and experiences of loneliness. However, in this population of young adults, social media emerges as the least powerful correlate of young people's reported loneliness, compared to other established risk factors such as bullying.^28^

## AUTHOR CONTRIBUTIONS

TM, LA, HLF, TEM, and CLO were involved in the conceptualization of the study. LA, TEM, and CLO obtained funding for the collection of the data used in the study. RG and JH were involved in the data collection. TM conducted the data analyses, interpreted the data, and drafted the initial manuscript. BTB reviewed the data analyses. All authors reviewed and approved the final manuscript prior to submission. The corresponding author confirms all listed authors meet the criteria for authorship.

## COMPETING INTERESTS

The authors declare no competing interests.

## PEER REVIEW

The peer review history for this article is available at https://publons.com/publon/10.1111/nyas.15370.

## Supporting information




**Figure S1**: Distribution of E‐Risk Study families’ home addresses across England and Wales
**Figure S2**: The E‐Risk Study families’ addresses are a near‐perfect match to the deciles of the UK Government's Index of Multiple Deprivation. This histogram shows E‐Risk families’ addresses are a near‐perfect match to the deciles of the UK's 2015 Lower‐layer Super Output Area (LSOA) Index of Multiple Deprivation (IMD) which averages 1500 residents; approximately 10% (dotted red line) of the ERisk cohort fills each of the IMD's 10% bands, indicating that the E‐Risk cohort accurately represents the distribution of deprivation in the UK. Note. The UK Ministry of Housing, Communities & Local Government Index of Multiple Deprivation is an official measure of relative deprivation for every LSOA small area (approximately 1500 residents or 650 households each) in England.
**Figure S3**: Timeline of recruitment to the SM2 study and changes to UK lockdown rules during the COVID‐19 pandemic.

Supporting Information

Supporting Information

Supporting Information

Supporting Information

Supporting Information

Supporting Information

Supporting Information

Supporting Information

## Data Availability

The data that support the findings of this study are not publicly available but can be accessed with permission from the E‐Risk Study team: https://eriskstudy.com/data‐access/
