# Supplementary material for: Social media use, online experiences, and loneliness among young adults: A cohort study
Source: Ann N Y Acad Sci. 2025 May 11;1548(1):194–205. doi: 10.1111/nyas.15370 (PMC12220285; doi:10.1111/nyas.15370)
Supplement: Supplementary file 1 — Figure S1: Distribution of E‐Risk Study families’ home addresses across England and Wales Figure S2: The E‐Risk Study families’ addresses are a near‐perfect match to the deciles of the UK Government's Index of Multiple Deprivation. This histogram shows E‐Risk families’ addresses are a near‐perfect match to the deciles of the UK's 2015 Lower‐layer Super Output Area (LSOA) Index of Multiple Deprivation (IMD) which averages 1500 residents; approximately 10% (dotted red line) of the ERisk cohort fills each of the IMD's 10% bands, indicating that the E‐Risk cohort accurately represents the distribution of deprivation in the UK. Note. The UK Ministry of Housing, Communities & Local Government Index of Multiple Deprivation is an official measure of relative deprivation for every LSOA small area (approximately 1500 residents or 650 households each) in England. Figure S3: Timeline of recruitment to the SM2 study and changes to UK lockdown rules during the COVID‐19 pandemic. [file NYAS-1548-194-s001.docx]

**SUPPORTING MATERIALS**

Problematic technology use scale

*In the past 12 months, how often have these things happened to you?*

- Found it difficult to stop using technology, such as the internet or your mobile phone, once you start?
- Short of sleep due to being on your phone or the internet late at night?
- Neglect your daily obligations (work, family, friends) because you are using technology?
- Feel restless, frustrated, or irritated when you cannot access the internet or check your phone?
- Use technology to escape from your sorrow or get relief from negative feelings?
- Choose to spend more time online over going out with others?
- People in your life complain about you spending too much time online?

*Response choices: Never (1), Sometimes (2), Often (3)*
