# Supplementary material for: Social media use, online experiences, and loneliness among young adults: A cohort study
Source: Ann N Y Acad Sci. 2025 May 11;1548(1):194–205. doi: 10.1111/nyas.15370 (PMC12220285; doi:10.1111/nyas.15370)
Supplement: Supplementary file 2 — Supporting Information [file NYAS-1548-194-s003.docx]

Supporting Table S1: Inter-correlations between reported time spent using different types of digital media.

|  | Social media | Watching TV | Gaming | Looking for information | Total time spent online |
| --- | --- | --- | --- | --- | --- |
| Social media | 1 |  |  |  |  |
| Watching TV | 0.32 | 1 |  |  |  |
| Gaming | 0.09 | 0.29 | 1 |  |  |
| Looking for information | 0.22 | 0.23 | 0.17 | 1 |  |
| Total time spent online | 0.4 | 0.35 | 0.26 | 0.42 | 1 |

All correlations are significant at the p < .001 level
