# Supplementary material for: Social media use, online experiences, and loneliness among young adults: A cohort study
Source: Ann N Y Acad Sci. 2025 May 11;1548(1):194–205. doi: 10.1111/nyas.15370 (PMC12220285; doi:10.1111/nyas.15370)
Supplement: Supplementary file 3 — Supporting Information [file NYAS-1548-194-s009.docx]

Supporting Table S2: Co-occurrence matrix of digital media platforms used by participants

|  | Facebook | WhatsApp | Instagram | YouTube | Snapchat | Twitter | Reddit | Dating sites/apps |
| --- | --- | --- | --- | --- | --- | --- | --- | --- |
| Facebook | 88% |  |  |  |  |  |  |  |
| WhatsApp | 77% | 87% |  |  |  |  |  |  |
| Instagram | 71% | 71% | 78% |  |  |  |  |  |
| YouTube | 64% | 64% | 56% | 73% |  |  |  |  |
| Snapchat | 51% | 51% | 49% | 40% | 56% |  |  |  |
| Twitter | 29% | 29% | 28% | 26% | 20% | 32% |  |  |
| Reddit | 11% | 11% | 9% | 12% | 6% | 5% | 13% |  |
| Dating sites/apps | 11% | 11% | 10% | 10% | 7% | 6% | 2% | 12% |

Percentages reflect the proportion of respondents who reported using the platforms in the intersecting rows and columns. For example; 88% of participants reported using Facebook, and 77% of respondents reported using both WhatsApp and Facebook. Proportions are calculated from participants with complete data for all variables (N = 1,629).
