# Supplementary material for: Social media use, online experiences, and loneliness among young adults: A cohort study
Source: Ann N Y Acad Sci. 2025 May 11;1548(1):194–205. doi: 10.1111/nyas.15370 (PMC12220285; doi:10.1111/nyas.15370)
Supplement: Supplementary file 4 — Supporting Information [file NYAS-1548-194-s008.docx]

Supporting Table S3: Inter-correlations between reported time spent using different digital media platforms

|  | Facebook | WhatsApp | Instagram | YouTube | Snapchat | Twitter | Reddit | Dating sites/apps |
| --- | --- | --- | --- | --- | --- | --- | --- | --- |
| Facebook | 1 |  |  |  |  |  |  |  |
| WhatsApp | 0.04 | 1 |  |  |  |  |  |  |
| Instagram | 0.21 *** | 0.27 *** | 1 |  |  |  |  |  |
| YouTube | 0.11 *** | 0.03 | 0.04 | 1 |  |  |  |  |
| Snapchat | 0.33 *** | 0.13 *** | 0.20 *** | 0.10 ** | 1 |  |  |  |
| Twitter | 0.06 | 0.02 | 0.21 *** | 0.19 *** | 0.16 ** | 1 |  |  |
| Reddit | -0.02 | -0.08 | 0.03 | 0.14 * | 0.00 | 0.09 | 1 |  |
| Dating sites/apps | 0.08 | -0.01 | 0.09 | 0.20 ** | 0.14 | 0.07 | 0.07 | 1 |

* p < 0.05, ** p < 0.01, *** p < 0.001. Pairwise N’s range from 35 (Dating sites/apps; Reddit) to 1,430 (WhatsApp; Facebook)
