# Supplementary material for: Social media use, online experiences, and loneliness among young adults: A cohort study
Source: Ann N Y Acad Sci. 2025 May 11;1548(1):194–205. doi: 10.1111/nyas.15370 (PMC12220285; doi:10.1111/nyas.15370)
Supplement: Supplementary file 5 — Supporting Information [file NYAS-1548-194-s005.docx]

Supporting Table S4: Demographics of study participants who took part before versus during the COVID-19 pandemic.

|  | **Before pandemic** | | **During pandemic** | | **Overall** | |
| --- | --- | --- | --- | --- | --- | --- |
|  | **%** | **N** | **%** | **N** | **%** | **N** |
| **Biological sex** |  |  |  |  |  |  |
| Male | 383 | 39.0% | 308 | 47.3% | 691 | 42.3% |
| Female | 598 | 61.0% | 343 | 52.7% | 941 | 57.7% |
| **Family SES** |  |  |  |  |  |  |
| Low | 281 | 28.6% | 232 | 35.6% | 513 | 31.4% |
| Middle | 304 | 31.0% | 223 | 34.3% | 527 | 32.3% |
| High | 396 | 40.4% | 196 | 30.1% | 592 | 36.3% |

N = Number. SES = Socioeconomic status.
