# Supplementary material for: Social media use, online experiences, and loneliness among young adults: A cohort study
Source: Ann N Y Acad Sci. 2025 May 11;1548(1):194–205. doi: 10.1111/nyas.15370 (PMC12220285; doi:10.1111/nyas.15370)
Supplement: Supplementary file 6 — Supporting Information [file NYAS-1548-194-s007.docx]

Supporting Table S5: Associations between depression and anxiety at age 18 and social media usage at age 26.

|  | **Association with:** | | | | | | | | | |
| --- | --- | --- | --- | --- | --- | --- | --- | --- | --- | --- |
|  | **Age-18 Major Depressive Disorder** | | | | | **Age-18 Generalised Anxiety Disorder** | | | | |
|  | **B** | **95% CI** | **β** | **95% CI** | **p** | **B** | **95% CI** | **β** | **95% CI** | **p** |
| **Time spent on types of digital media:** |  |  |  |  |  |  |  |  |  |  |
| Social media | 0.18 | -0.02, 0.38 | 0.05 | -0.01, 0.10 | 0.080 | -0.06 | -0.36, 0.25 | -0.01 | -0.06, 0.04 | 0.709 |
| Watching TV | 0.12 | -0.07, 0.31 | 0.03 | -0.02, 0.08 | 0.213 | 0.19 | -0.10, 0.49 | 0.03 | -0.02, 0.08 | 0.203 |
| Gaming | 0.16 | -0.07, 0.40 | 0.03 | -0.01, 0.08 | 0.166 | 0.08 | -0.24, 0.41 | 0.01 | -0.03, 0.05 | 0.614 |
| Looking for information | 0.37 | 0.16, 0.58 | 0.09 | 0.04, 0.14 | **0.001** | 0.30 | -0.02, 0.62 | 0.05 | 0.00, 0.10 | 0.068 |
| Total time online | 0.24 | 0.07, 0.41 | 0.07 | 0.02, 0.11 | **0.007** | 0.18 | -0.08, 0.44 | 0.03 | -0.01, 0.08 | 0.172 |
| **Time spent on specific platforms:** |  |  |  |  |  |  |  |  |  |  |
| Facebook | -0.04 | -0.08, 0.01 | -0.04 | -0.10, 0.01 | 0.098 | -0.04 | -0.10, 0.03 | -0.03 | -0.08, 0.02 | 0.273 |
| WhatsApp | -0.03 | -0.08, 0.01 | -0.04 | -0.09, 0.01 | 0.113 | 0.02 | -0.03, 0.08 | 0.02 | -0.02, 0.06 | 0.361 |
| Instagram | -0.01 | -0.06, 0.04 | -0.01 | -0.06, 0.04 | 0.734 | -0.06 | -0.14, 0.03 | -0.03 | -0.09, 0.19 | 0.208 |
| YouTube | 0.09 | 0.04, 0.14 | 0.08 | 0.04, 0.13 | **< 0.001** | 0.09 | 0.02, 0.17 | 0.05 | 0.01, 0.10 | **0.017** |
| Snapchat | -0.01 | -0.07, 0.05 | -0.01 | -0.06, 0.04 | 0.829 | -0.06 | -0.15, 0.04 | -0.03 | -0.08, 0.19 | 0.229 |
| Twitter | -0.18 | -0.07, 0.04 | -0.02 | -0.06, 0.03 | 0.541 | 0.04 | -0.05, 0.12 | 0.02 | -0.03, 0.07 | 0.426 |
| Reddit | 0.05 | 0.00, 0.09 | 0.05 | 0.00, 0.10 | **0.033** | 0.07 | 0.00, 0.14 | 0.06 | -0.00, -0.11 | **0.037** |
| Dating sites/apps | 0.01 | -0.33, 0.05 | 0.01 | -0.04, 0.06 | 0.747 | 0.08 | -.01, 0.16 | 0.07 | 0.01, 0.13 | **0.021** |

B = unstandardised regression coefficient. β = standardised regression coefficient. CI = confidence interval. All analyses are adjusted for sex, socioeconomic status, and the non-independence of twin observations. Significant p-values are indicated in bold.
