# Supplementary figures and images for: Social media use, online experiences, and loneliness among young adults: A cohort study
Source: Ann N Y Acad Sci. 2025 May 11;1548(1):194–205. doi: 10.1111/nyas.15370 (PMC12220285; doi:10.1111/nyas.15370)

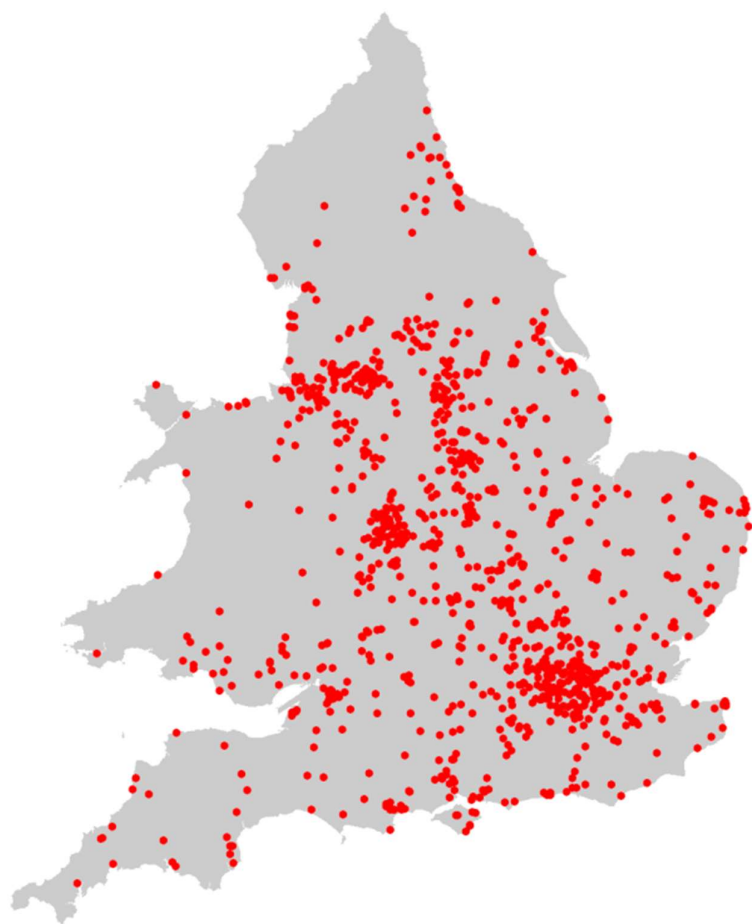

Supplement: Supplementary file 7 — Supporting Information [file NYAS-1548-194-s002.pdf]

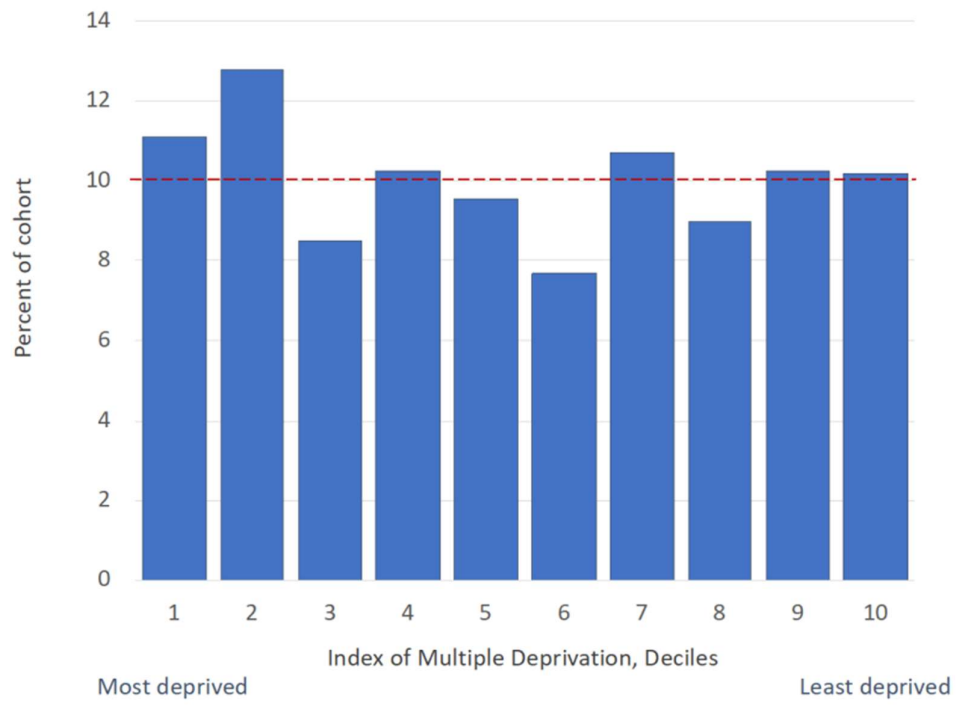

Supplement: Supplementary file 8 — Supporting Information [file NYAS-1548-194-s006.pdf]

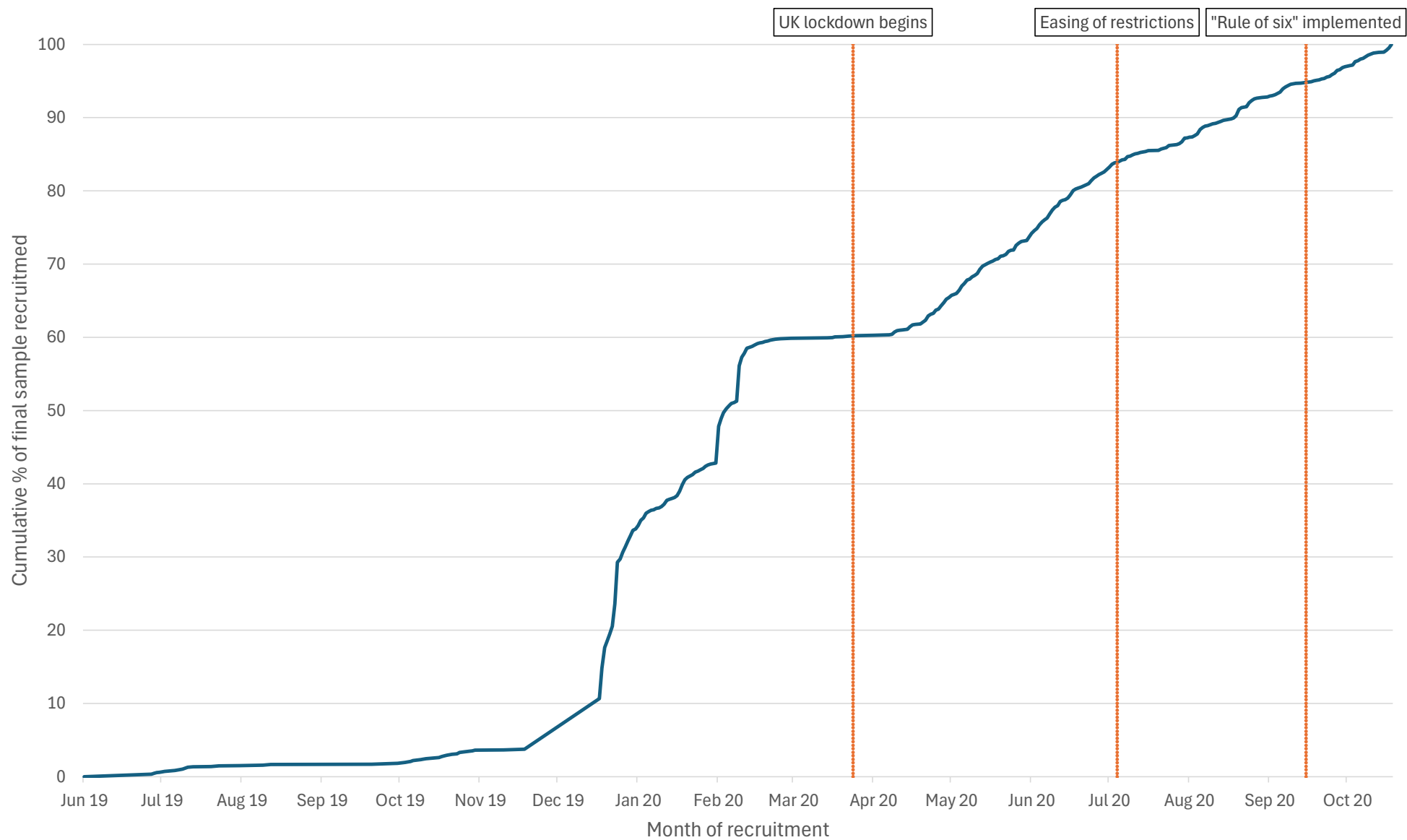

Supplement: Supplementary file 9 — Supporting Information [file NYAS-1548-194-s004.pdf]
